# Co-Designing the ADRD Systematic Hospital Inclusion Family Toolkit (ASHIFT)

**DOI:** 10.1093/geroni/igaf122.040

**Published:** 2025-12-31

**Authors:** Nicole Werner, Sydney Hoel, Teresa Thuemling, Linda Haack, Beth Fields

**Affiliations:** Vanderbilt University Medical Center, Nashville, Tennessee, United States; Vanderbilt University Medical Center, Nashville, Tennessee, United States; Vanderbilt University Medical Center, Nashville, Tennessee, United States; Caregiver Partner, Nasvhille, Tennessee, United States; University of Wisconsin–Madison, Madison, Wisconsin, United States

## Abstract

Care partners (family or friends) of people living with Alzheimer’s disease and related dementias (ADRD) are often not systematically included when the person with ADRD is hospitalized. This lack of care partner inclusion is associated with suboptimal outcomes for both ADRD patients and their care partners. Research and policy call for care partner inclusion in hospitalizations, but no tools exist to guide healthcare systems on care partner inclusion. This study aimed to co-design ASHIFT, an adaptable toolkit to be used by healthcare systems to facilitate the inclusion of care partners of hospitalized people living with ADRD. We convened two co-design groups – one of care partners of people living with ADRD who experienced a hospitalization (n = 6) and one of clinicians who care for patients living with ADRD (n = 5). We conducted five parallel co-design sessions over Zoom. Across the sessions, co-designers created ASHIFT, starting with many ideas and converging on the final prototype. ASHIFT provides guidance on how to identify, assess, and train care partners of hospitalized people living with ADRD and resources to support each action. Guidance is divided into hospitalization stages, with specific guidance for admission, hospital stay, discharge, and post-discharge. Each stage provides specific assets and protocols and links to resources. To support implementation, ASHIFT also provides an organizational readiness assessment, suggestions on prioritizing actions, and which actions can be conducted remotely. The next step is to test the feasibility and effect of ASHIFT on increasing care partner preparedness and reducing avoidable hospital readmission for patients with ADRD.

